# Effects of Gut Microbiota-Modulating Interventions on Systemic Inflammation and Functional Outcomes in Established Cardiovascular Disease: A Systematic Review

**DOI:** 10.7759/cureus.107856

**Published:** 2026-04-28

**Authors:** Ishtiaq Ahmad, Manoj Sivakumar, Shivam Singla, Bhavna Singla, Sunita Kumawat, Ghazanfar Ali, Ali Raza Mehdi

**Affiliations:** 1 Internal Medicine, University of Iowa Hospitals and Clinics, Iowa City, USA; 2 Internal Medicine, K.A.P. Viswanatham Government Medical College, Tiruchirappalli, IND; 3 Internal Medicine, TidalHealth Peninsula Regional, Salisbury, USA; 4 Internal Medicine, Erie County Medical Center, Buffalo, USA; 5 Internal Medicine, St. Francis Medical Center, Lynwood, USA; 6 Internal Medicine, Medicare Hospital, Faisalabad, PAK; 7 Internal Medicine, Services Hospital Lahore, Lahore, PAK

**Keywords:** cardiovascular disease, coronary artery disease, gut microbiota, heart failure, inflammation, metabolic endotoxemia, postbiotics, probiotics, randomized controlled trial, stroke

## Abstract

Cardiovascular disease remains a leading cause of morbidity and mortality worldwide, with persistent residual inflammatory risk despite advances in contemporary pharmacotherapy. Growing evidence implicates the gut-heart axis in the pathogenesis of atherosclerosis, heart failure, and cerebrovascular disease through mechanisms involving dysbiosis, intestinal permeability, endotoxemia, and systemic inflammation. This systematic review aimed to evaluate the effects of gut microbiota-modulating interventions on systemic inflammatory markers and functional or clinical outcomes in adults with established cardiovascular disease. A comprehensive search of PubMed/Medical Literature Analysis and Retrieval System Online (MEDLINE), Scopus, and Web of Science was conducted for randomized controlled trials published between January 2020 and December 2024. Eligible studies included adult patients with documented coronary artery disease, heart failure, or cerebrovascular accident receiving probiotics, postbiotics, or microbiota-targeted antibiotics compared with a placebo or standard care. Four randomized clinical trials met the inclusion criteria. Across studies, microbiota-modulating interventions were associated with significant reductions in inflammatory biomarkers, including interleukin-1β, high-sensitivity C-reactive protein, and lipopolysaccharide, suggesting attenuation of metabolic endotoxemia and systemic inflammatory activity. Improvements in functional capacity were observed in one chronic heart failure trial, whereas a multicenter heart failure study did not demonstrate significant changes in ventricular function or inflammatory markers. Overall, the available evidence indicates that microbiota-targeted interventions may exert anti-inflammatory effects in established cardiovascular disease; however, consistent functional or structural cardiac benefits have not been demonstrated. The current literature is limited by small sample sizes, heterogeneous interventions, and short follow-up durations. Larger, adequately powered trials with standardized endpoints are needed to determine whether modulation of the gut microbiota can translate into meaningful clinical benefit in secondary cardiovascular prevention.

## Introduction and background

Cardiovascular disease remains the leading cause of morbidity and mortality worldwide, encompassing conditions such as coronary artery disease, heart failure, and cerebrovascular disease. Despite advances in pharmacotherapy, interventional cardiology, and secondary prevention strategies, a substantial residual risk persists in many patients with established cardiovascular disease [1,2]. Chronic low-grade systemic inflammation has emerged as a central pathophysiological mechanism underlying atherosclerosis progression, plaque instability, adverse ventricular remodeling, and recurrent ischemic events. Circulating inflammatory biomarkers such as high-sensitivity C-reactive protein, interleukin-6, and tumor necrosis factor alpha have been consistently associated with disease severity and clinical outcomes across cardiovascular phenotypes [3].

In recent years, increasing attention has been directed toward the gut-heart axis as a potential contributor to cardiovascular pathogenesis. The intestinal microbiota influences host metabolism, immune regulation, and barrier integrity. Alterations in gut microbial composition and function, often described as dysbiosis, have been linked to enhanced intestinal permeability, microbial translocation, and the generation of bioactive metabolites such as trimethylamine N-oxide [4,5]. These processes may promote endothelial dysfunction, vascular inflammation, and myocardial remodeling. Observational studies have demonstrated differences in microbial profiles in patients with heart failure, coronary artery disease, and stroke compared with healthy controls, suggesting a potential mechanistic link between the gut microbiome and established cardiovascular disease [6].

Interventions aimed at modulating the gut microbiota have therefore been proposed as adjunctive therapeutic strategies. These approaches include probiotics, prebiotics, synbiotics, postbiotics, dietary fiber-based interventions, and microbiota-targeted antibiotics. Experimental and early-phase clinical studies suggest that such interventions may attenuate systemic inflammation, reduce endotoxemia, and favorably influence metabolic and vascular parameters. However, the translation of these findings into patients with established cardiovascular disease remains uncertain [7]. The available randomized clinical trials are limited in number and vary in terms of population, intervention type, duration, and measured outcomes. Furthermore, while inflammatory biomarkers are frequently reported, data on functional outcomes and clinically meaningful endpoints are less consistently evaluated [8].

Given the growing interest in microbiota-targeted therapies and the persistent inflammatory burden observed in patients with established cardiovascular disease, a critical appraisal of the current interventional evidence is warranted. Clarifying whether modulation of the gut microbiota can meaningfully influence systemic inflammation or functional status in this population is important for guiding future research and potential therapeutic development. The objective of this systematic review is to evaluate the effects of gut microbiota-modulating interventions, compared with placebo or standard care, on systemic inflammatory markers and functional or clinical outcomes in adults with established cardiovascular disease.

## Review

Materials and methods 

Study Design and Reporting Standards

This systematic review was conducted to evaluate the effects of gut microbiota-modulating interventions on systemic inflammatory markers and functional or clinical outcomes in adults with established cardiovascular disease. The methodology was developed in accordance with the Preferred Reporting Items for Systematic Reviews and Meta-Analyses (PRISMA) guidelines [9] to ensure transparent reporting and reproducibility. The review focused exclusively on randomized clinical trials to strengthen internal validity and minimize confounding inherent to observational designs.

Eligibility Criteria

The eligibility criteria were structured according to the Population, Intervention, Comparator, Outcomes, and Study Design framework [10]. The population of interest included adults aged 18 years or older with established cardiovascular disease, defined as documented coronary artery disease, heart failure, or cerebrovascular accident. Studies conducted in primary prevention populations or in patients with isolated cardiometabolic risk factors without confirmed cardiovascular disease were excluded. Eligible interventions comprised gut microbiota-modulating strategies, including probiotics, postbiotics, synbiotics, microbiota-targeted antibiotics, or related dietary interventions specifically intended to alter gut microbial composition or function. Comparators included placebo, standard of care, or control interventions. Primary outcomes of interest were systemic inflammatory biomarkers, such as interleukin-1β, high-sensitivity C-reactive protein, tumor necrosis factor alpha, lipopolysaccharide, or related markers of endotoxemia and oxidative stress. Secondary outcomes included functional or clinical endpoints, such as measures of exercise capacity, ventricular function, or validated clinical severity scores. Only randomized controlled trials published in peer-reviewed journals were included.

Information Sources and Search Strategy

A comprehensive literature search was conducted in three electronic databases, including PubMed/Medical Literature Analysis and Retrieval System Online (MEDLINE), Scopus, and Web of Science. The search was restricted to studies published within the last five years, from January 2020 through December 2024, to ensure inclusion of contemporary randomized evidence reflecting current understanding of the gut-heart axis. The search strategy incorporated both controlled vocabulary and free-text terms. In PubMed, Medical Subject Headings (MeSH) such as “Cardiovascular Diseases,” “Heart Failure,” “Coronary Artery Disease,” “Stroke,” “Gastrointestinal Microbiome,” “Probiotics,” “Anti-Bacterial Agents,” and “Inflammation” were used. These were combined with keywords including gut microbiota, dysbiosis, postbiotic, synbiotic, endotoxemia, lipopolysaccharide, C-reactive protein, interleukin, and functional capacity. Boolean operators were applied to structure the search strategy, using combinations such as (“Cardiovascular Diseases” OR “Heart Failure” OR “Coronary Artery Disease” OR “Stroke”) AND (“Gastrointestinal Microbiome” OR probiotic* OR postbiotic* OR synbiotic* OR rifaximin) AND (inflammation OR endotoxin OR “C-reactive protein” OR interleukin OR “functional capacity” OR “exercise tolerance”). Truncation symbols were used where appropriate to capture variations of key terms. Filters were applied to limit results to randomized controlled trials and articles published in English. No geographic restrictions were imposed. Reference lists of eligible studies were manually screened to identify additional relevant trials.

Study Selection

Titles and abstracts identified through the search were screened for relevance. Full texts of potentially eligible studies were retrieved and assessed against predefined inclusion and exclusion criteria. Discrepancies in study selection were resolved through discussion to ensure consistent application of eligibility criteria. The selection process followed the PRISMA framework, documenting the number of records identified, screened, excluded, and included in the final analysis.

Data Extraction

Data were extracted using a predefined standardized extraction form tailored to the objectives of this review. Extracted variables included study characteristics, sample size, cardiovascular disease subtype, participant demographics, intervention type and duration, comparator details, inflammatory biomarkers assessed, functional or clinical outcomes measured, and main findings. Particular attention was given to the measurement and reporting of systemic inflammatory markers and functional endpoints to ensure alignment with the review question. Extracted data were verified for accuracy and completeness prior to synthesis.

Risk of Bias Assessment

Methodological quality was evaluated using the Cochrane Risk of Bias 2 (RoB 2) tool [11] for randomized trials. Domains assessed included bias arising from the randomization process, deviations from intended interventions, missing outcome data, measurement of outcomes, and selection of reported results. Each study was classified as having a low risk of bias or some concerns based on domain-level judgments. This structured appraisal enabled a transparent assessment of internal validity across included trials.

Data Synthesis

Given the limited number of eligible trials and the heterogeneity in intervention types, patient populations, and reported endpoints, a quantitative meta-analysis was not performed. Instead, a qualitative narrative synthesis was conducted. Findings were interpreted in relation to cardiovascular subtype, type of microbiota-modulating intervention, duration of therapy, and measured outcomes. The synthesis emphasized consistency and divergence across studies, with particular focus on systemic inflammatory modulation and functional or clinical effects in established cardiovascular disease. This methodological approach was designed to provide a rigorous and focused evaluation of interventional evidence targeting the gut-heart axis in secondary cardiovascular prevention populations.

Results

Study Selection Process

**Figure 1:**
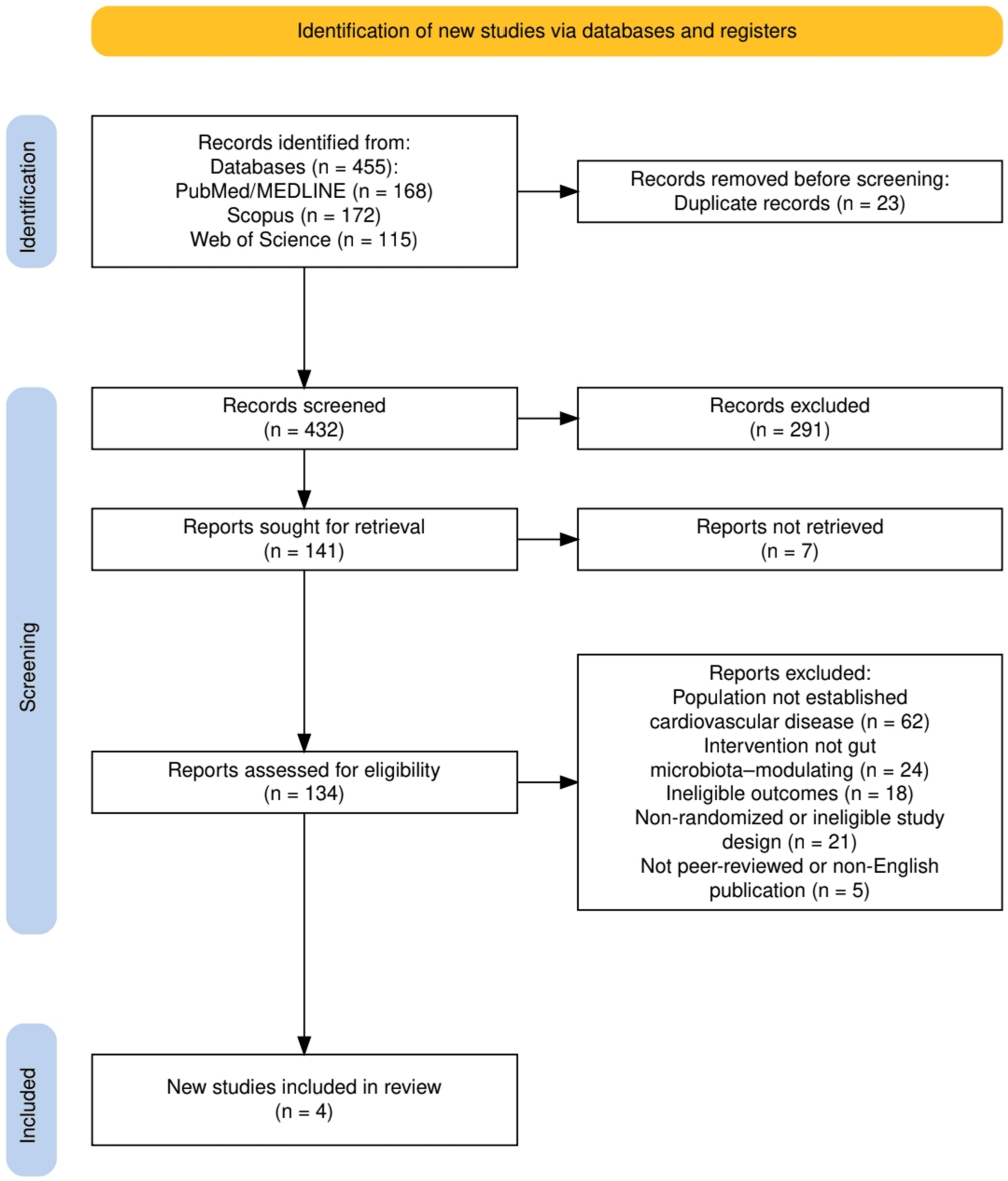
The PRISMA flow diagram represents the study selection process. PRISMA: Preferred Reporting Items for Systematic reviews and Meta-Analyses; MEDLINE: Medical Literature Analysis and Retrieval System Online

The study selection process followed the PRISMA framework and is illustrated in Figure 1. A total of 455 records were identified through database searching, including PubMed/MEDLINE, Scopus, and Web of Science. After the removal of 23 duplicate records, 432 titles and abstracts were screened for relevance. Of these, 291 records were excluded based on predefined eligibility criteria. A total of 141 full-text articles were sought for retrieval, of which seven could not be accessed. The remaining 134 reports underwent full-text assessment for eligibility. Following detailed evaluation using the Population, Intervention, Comparator, Outcomes, and Study Design framework, 130 studies were excluded for reasons including non-established cardiovascular populations, ineligible interventions or outcomes, non-randomized design, or publication-related criteria. Ultimately, four randomized clinical trials met all inclusion criteria and were included in the final qualitative synthesis.

Characteristics of the Selected Studies

The characteristics of the included randomized clinical trials are summarized in Table 1. A total of four trials involving patients with established cardiovascular disease were included, encompassing populations with chronic heart failure, coronary artery disease, and cerebrovascular accident. Sample sizes ranged from 44 to 151 participants, with intervention durations varying between seven days and three months. The evaluated interventions included multistrain probiotics, a single-strain probiotic, postbiotic supplementation, and a microbiota-targeted antibiotic, each compared with placebo or standard care. All studies assessed systemic inflammatory biomarkers, including interleukin-1β, high-sensitivity C-reactive protein, lipopolysaccharide, and related markers of endotoxemia or oxidative stress. Functional or clinical outcomes were variably reported and included measures of physical performance, ventricular function, and clinical severity scores. Overall, the included studies reflect methodological diversity in intervention type and outcome selection within the context of secondary cardiovascular prevention.

**Table 1:** Characteristics of randomized controlled trials evaluating gut microbiota-modulating interventions in established cardiovascular disease. CVD: cardiovascular disease; CHF: chronic heart failure; HFrEF: heart failure with reduced ejection fraction; CAD: coronary artery disease; CVA: cerebrovascular accident; IL-1β: interleukin-1 beta; hs-CRP: high-sensitivity C-reactive protein; LPS: lipopolysaccharide; CRP: C-reactive protein; TMAO: trimethylamine N-oxide; LVEF: left ventricular ejection fraction; SPPB: Short Physical Performance Battery; NIHSS: National Institutes of Health Stroke Scale; APACHE II: Acute Physiology and Chronic Health Evaluation II; NUTRIC: Nutrition Risk in the Critically Ill; CFU: colony-forming units; RCT: randomized controlled trial

Study (Author, Year)	CVD Population	Sample Size (I/C)	Intervention (Type & Duration)	Comparator	Primary Inflammatory Outcomes	Functional / Clinical Outcomes	Main Findings
Karim et al., 2022 [12]	Chronic heart failure (CHF)	44 / 48	Multistrain probiotic, 12 weeks	Placebo	Zonulin, Dkk-1, Dkk-3, SREBP1; inflammatory and oxidative stress markers	Handgrip strength, gait speed, SPPB score	Improved functional capacity and reduced inflammatory and intestinal permeability markers; Wnt biomarker modulation associated with reduced sarcopenia
Rahimi et al., 2024 [13]	Cerebrovascular accident (CVA)	60 / 60	Postbiotic supplementation, 7 days	Placebo	IL-1β, hs-CRP, MDA, TAC	Pneumonia incidence; NIHSS, APACHE II, NUTRIC scores	Significant reduction in inflammatory and oxidative stress markers; reduced pneumonia incidence; no improvement in neurological severity scores
Moludi et al., 2021 [14]	Coronary artery disease (CAD)	22 / 22 (total 44)	Lactobacillus rhamnosus GG (1.6 × 10⁹ CFU/day), 12 weeks (with calorie restriction)	Placebo + calorie restriction	IL-1β, LPS, TLR4, IL-10	Cardiovascular risk markers (no direct functional exercise assessment)	Significant reduction in IL-1β and LPS levels; improvement in metabolic endotoxemia in CAD patients
Awoyemi et al., 2021 (GutHeart Trial) [15]	Heart failure with reduced ejection fraction (HFrEF)	151 total (3 arms: rifaximin, Saccharomyces boulardii, SoC)	Probiotic yeast or rifaximin, 3 months	Standard of care	CRP, TMAO, microbiota diversity	Left ventricular ejection fraction (LVEF)	No significant effect on LVEF, CRP, TMAO, or microbiota diversity compared with standard care

Quality Assessment

The methodological quality of the included studies was assessed using the Cochrane RoB 2 tool, and the results are summarized in Table 2. Two trials were judged to have an overall low risk of bias, reflecting appropriate randomization procedures, adequate blinding, and objective outcome assessment. The remaining two studies were categorized as having some concerns, primarily related to limitations in reporting of allocation concealment or the open-label design with blinded endpoint assessment. No study was considered to be at high risk of bias. Overall, the included trials demonstrated acceptable methodological rigor, although certain domains warrant cautious interpretation when considering the strength and generalizability of the findings.

**Table 2:** Risk of bias assessment of randomized controlled trials evaluating microbiota-modulating interventions in cardiovascular disease. RoB 2: Cochrane Risk of Bias 2 tool; RCT: randomized controlled trial; CVD: cardiovascular disease; LVEF: left ventricular ejection fraction

Study (Author, Year)	Study Design Features	Overall Risk of Bias (RoB 2)	Rationale
Karim et al., 2022 [12]	Randomized, placebo-controlled trial; blinding details limited in abstract	Some concerns	Randomization reported, but allocation concealment and detailed blinding procedures not fully described in accessible report; small sample size
Rahimi et al., 2024 [13]	Randomized, controlled trial with reported blinding	Low risk	Clear randomization, placebo control, defined outcomes, and short intervention period with objective biomarker assessment
Moludi et al., 2021 [14]	Double-blind, placebo-controlled RCT	Low risk	Explicit double-blind design, objective laboratory biomarkers, appropriate comparator, and clearly defined intervention protocol
Awoyemi et al., 2021 (GutHeart Trial) [15]	Multicenter randomized open-label trial with blinded endpoint assessment	Some concerns	Randomized with blinded outcome assessment, but open-label treatment allocation introduces potential performance bias

Discussion

This systematic review evaluated the effects of gut microbiota-modulating interventions on systemic inflammation and functional outcomes in adults with established cardiovascular disease. Across four randomized clinical trials, microbiota-targeted strategies demonstrated consistent signals toward inflammatory modulation, particularly in coronary artery disease and cerebrovascular populations. Moludi et al. [14] reported reductions in IL-1β and lipopolysaccharide levels in patients with coronary artery disease, while Rahimi et al. [13] observed significant decreases in IL-1β, high-sensitivity C-reactive protein, and oxidative stress markers in patients with acute stroke. In chronic heart failure, Karim et al. [12] demonstrated improvements in functional capacity alongside reductions in inflammatory and intestinal permeability markers. In contrast, the multicenter GutHeart trial by Awoyemi et al. [15] did not show significant improvements in left ventricular ejection fraction or inflammatory biomarkers following probiotic or rifaximin therapy. Overall, the available evidence suggests potential anti-inflammatory effects of microbiota-modulating interventions, although functional and structural cardiac benefits remain inconsistent and the evidence base is limited in size and heterogeneity.

The observed findings can be interpreted within the framework of the gut-heart axis. Dysbiosis may contribute to increased intestinal permeability, microbial translocation, and metabolic endotoxemia, which in turn promote systemic inflammation, endothelial dysfunction, and myocardial remodeling. The reductions in IL-1β and lipopolysaccharide reported by Moludi et al. [14] and Rahimi et al. [13] are consistent with attenuation of this inflammatory cascade, supporting a mechanistic link between gut-derived signals and cardiovascular inflammation. In chronic heart failure, Karim et al. [12] demonstrated modulation of Wnt signaling biomarkers alongside improvements in muscle performance, suggesting a possible interaction between gut dysbiosis, inflammation, and skeletal muscle function in this population. Conversely, the neutral findings of Awoyemi et al. [15] indicate that short-term modulation of microbiota composition or metabolites may not be sufficient to reverse established structural cardiac dysfunction. Taken together, these results suggest that reductions in inflammatory biomarkers may precede measurable changes in cardiac structure or functional capacity and that intervention duration and disease stage likely influence therapeutic responsiveness.

The variability in findings across the included trials likely reflects important clinical and methodological heterogeneity. The study populations differed substantially, ranging from stable coronary artery disease in Moludi et al. [14] to acute cerebrovascular events in Rahimi et al. [13] and chronic heart failure in Karim et al. [12] and Awoyemi et al. [15]. These conditions represent distinct stages and phenotypes of cardiovascular disease, with differing inflammatory burdens and structural remodeling profiles. Intervention strategies also varied, including strain-specific probiotics, postbiotic supplementation, and microbiota-targeted antibiotic therapy, each with potentially different biological effects. The duration of treatment ranged from seven days in acute stroke to 12 weeks and three months in chronic conditions, which may influence the capacity to observe downstream structural or functional changes [16-18]. Furthermore, primary endpoints differed across studies, with some focusing primarily on inflammatory biomarkers and others on ventricular function or physical performance measures. Collectively, these differences suggest that microbiota-targeted therapy may exert more discernible effects in inflammation-dominant states, such as acute stroke or metabolic endotoxemia, than in advanced structural heart failure, where myocardial remodeling is already established [19].

The reductions in IL-1β observed in coronary artery disease and stroke populations align with contemporary understanding of inflammation-driven atherothrombosis, particularly in the context of interleukin-1 signaling as highlighted in the CANTOS (Canakinumab Anti-Inflammatory Thrombosis Outcomes Study) era [20,21]. Decreases in lipopolysaccharide levels, as reported by Moludi et al. [14], suggest attenuation of metabolic endotoxemia and reduced activation of Toll-like receptor-mediated inflammatory pathways. In chronic heart failure, the modulation of Wnt signaling biomarkers described by Karim et al. [12] may reflect a complex interaction between intestinal dysbiosis, systemic inflammation, and skeletal muscle metabolism, supporting the concept of muscle-inflammation cross-talk in heart failure-associated sarcopenia. Conversely, the absence of significant improvement in left ventricular ejection fraction in the GutHeart trial by Awoyemi et al. [15] indicates that short-term alteration of microbiota composition or metabolite levels may not be sufficient to reverse established ventricular remodeling. These findings situate the present review within the broader cardiology literature by suggesting that microbiota modulation may primarily influence inflammatory and metabolic pathways, with structural cardiac benefits requiring longer duration or multimodal therapeutic strategies.

Previous reviews examining the cardiovascular relevance of the gut microbiome have largely concentrated on metabolic syndrome, obesity, diabetes, or primary prevention cohorts, where cardiometabolic risk factors predominate but established cardiovascular disease is absent. In contrast, relatively few randomized clinical trials have evaluated microbiota-modulating interventions in patients with confirmed coronary artery disease, heart failure, or cerebrovascular events [22]. By restricting inclusion to secondary prevention populations and randomized designs, the present review addresses a narrower yet clinically more consequential question. The limited number of eligible trials underscores that the principal gap in the literature is not mechanistic plausibility, which is well supported by experimental and observational data, but rather the scarcity of adequately powered, methodologically rigorous interventional studies in patients with established cardiovascular disease [23].

The current body of evidence does not support the routine use of probiotics, postbiotics, or microbiota-targeted antibiotics as standard therapy in patients with established cardiovascular disease. Although reductions in inflammatory and endotoxemia-related biomarkers were observed in several trials, consistent improvements in structural cardiac parameters or validated functional outcomes were not demonstrated. Nonetheless, the anti-inflammatory signals reported suggest that microbiota-modulating strategies may have potential as adjunctive interventions, particularly in patients with heightened inflammatory profiles [24]. Future trials should be larger, strain-specific, and of sufficient duration to assess meaningful changes in ventricular remodeling, exercise capacity, hospitalization rates, and major adverse cardiovascular events. Prioritizing clinically relevant endpoints will be essential to determine whether modulation of the gut microbiota can translate into tangible cardiovascular benefit [25].

This review has several methodological strengths. It applied strict inclusion criteria, limiting eligibility to adults with established cardiovascular disease, thereby focusing on secondary prevention rather than broader cardiometabolic risk populations. Only randomized clinical trials were included, enhancing internal validity and minimizing confounding inherent to observational designs. The review specifically examined systemic inflammatory markers and functional or clinical outcomes, aligning closely with mechanistic and patient-centered endpoints relevant to cardiovascular disease progression. Importantly, both positive and neutral findings were reported without selective emphasis, providing a balanced synthesis of the available evidence and avoiding overinterpretation of preliminary signals.

The limitations of this review largely reflect the current state of the field. The number of eligible randomized trials in established cardiovascular disease remains small, and the included studies demonstrate heterogeneity in patient populations, intervention types, and selected endpoints. Intervention durations were relatively short, ranging from days to a few months, which may be insufficient to detect changes in structural cardiac remodeling or long-term clinical outcomes. Functional exercise capacity was not uniformly assessed across trials, limiting direct comparison of performance-based outcomes. The variability in study design and endpoints precluded quantitative synthesis, and a meta-analysis was therefore not feasible. These constraints underscore the early stage of interventional research targeting the gut-heart axis.

Future investigations should prioritize standardization of inflammatory and metabolic endpoints to facilitate cross-trial comparability and potential pooled analyses. Longer intervention durations are warranted to determine whether microbiota modulation can influence ventricular remodeling, recurrent ischemic events, or progression of heart failure. Integrating comprehensive microbiome sequencing with predefined clinical endpoints may clarify responder phenotypes and mechanistic pathways. Stratification based on baseline inflammatory burden, such as elevated high-sensitivity C-reactive protein or endotoxemia markers, may help identify subgroups most likely to benefit from targeted interventions. Additionally, combination strategies pairing microbiota modulation with established anti-inflammatory pharmacotherapy merit exploration. Rather than universal supplementation, future trials should consider an inflammatory phenotype-guided approach to optimize therapeutic precision within cardiovascular populations.

## Conclusions

Current randomized evidence suggests that gut microbiota-modulating interventions exert measurable anti-inflammatory effects in patients with established cardiovascular disease, particularly through reductions in interleukin-1β and endotoxemia-related markers. However, consistent improvements in cardiac structure or validated functional outcomes have not yet been demonstrated. The available trials indicate biological plausibility and early clinical signals, but they remain limited in scale, duration, and endpoint selection. The central implication is that modulation of the gut-heart axis holds therapeutic potential, yet it cannot presently be recommended as routine adjunctive therapy in secondary cardiovascular prevention. Advancing this field will require adequately powered, longer-duration trials with standardized inflammatory and clinically meaningful cardiovascular outcomes. Until such data are available, microbiota-targeted strategies should be regarded as promising but investigational within the context of established cardiovascular disease.
